# A Treatise on Appendicitis

**Published:** 1896-09

**Authors:** 


### BOOK NOTICES.

A TREATISE ON APPENDICITIS. By John B. Deaver, M. D., Surgeon to the German Hospital, Philadelphia. Containing 32 full-page plates and other illustrations. Philadelphia: P. Blakiston, Son & Co., 1012 Walnut Street. 1896. Price, cloth, $3.50.

At the present day, when so much interest has been aroused alike among the laity and the profession by the frequent occurrence of the comparatively newly-discovered disease, appendicitis, a masterly exposition of this source of human affliction is urgently needed. Such a treatise has now been placed before the profession, and is at present the object of this review.

The obscure character of appendicitis and the rapidity with which the disease runs its course and ends in death, makes it absolutely necessary that every physician should be perfectly familiar with its characteristics, that he may determine it at the earliest possible moment, and so decide upon the course to be pursued. The book now under notice fulfills the conditions for enabling the physician to become perfectly instructed in everything pertaining to the subject.

The author, a Philadelphia physician of high standing, has made it a special study, and has operated in over five hundred cases, and is therefore competent to give the desired information.

The author finds that the cæcum from which the appendix is a process occurs as one or other of four types. These are clearly illustrated by plates. Its exact position in relation to other organs, and especially the peritoneum, is clearly and minutely shown in plates and described in the text.

There are eight positions in which the appendix may be found. These are all carefully described, the positions being indicated by marking the figure of the abdomen with the points of the compass like a map. These positions are illustrated by drawings taken from dissections especially for this work. The reader's attention is next drawn to the question of the cause of appendicitis. He rejects the popular idea that it is always due to the seeds or pits of fruit being ensnared in the folds of this organ, though he admits that rarely such accidents may bring it about, and gives a remarkable plate of a case in his practice where a *pin* may be seen protruding through the walls of the appendix.

He thinks that most of the cases reported as owing to fruit pits are really fecal concretions, "which bear a very striking resemblance to such foreign bodies." These fecal concretions he considers the exciting cause of the disease in many instances, and holds that when first introduced into the lumen of the appendix they are very small, but being retained by the swelling of the lining membrane gradually grow larger by concentric layers of mucous and purulent products deposited upon their surface.

This accounts for the presence of calculi in the appendix which are too large to have entered it through the mouth of the organ.

It further appears that the appendix is furnished with circular muscular fibres by which it is endowed with a peristaltic motion by which it is enabled to empty itself of accumulations.

That the appendix should be so frequently involved in these attacks of inflammation with all their disastrous sequalæ is accounted for by an inquiry into the place it occupies in the history of evolution. "The appendix vermiformis of man is the undeveloped true cœcum of some of the lower animals" (page 23). It is, therefore, undergoing a retrograde metamorphosis in the process of evolution. It is furnished with a very limited blood supply and so is an organ of low vitality (page 31). Consequently its powers of resistance are decreased (page 70).

The predisposing causes in the author's opinion, are the anatomical structure and position of the appendix, catarrhal inflammation of the lining mucous membrane, age, sex, tuberculosis, exposure to cold and wet (such as a cold shower bath following a warm bath), typhoid fever and micro-organisms.

He thinks that the immediate cause is always an "invasion of micro-organisms." He is certain that "acute indigestion plays a very important *rôle* in the ætiology."

The pathology of appendicitis is given minutely and illustrated by several beautiful plates in colors, and this is followed by the symptomatology.

There are three symptoms so constant and so characteristic of appendicitis, that the author calls them the "The Three Cardinal Symptoms." They are: pain, tenderness upon pressure, and rigidity of the abdominal walls. The other symptoms of less importance are also mentioned.

The prognosis in appendicitis depends mainly upon the interval of time between the access of inflammation and the time of operation. This interval of time should not exceed twenty-four hours. The longer the interval the less are the chances of recovery.

The most minute directions far the operation are given and every step is illustrated by a beautiful colored plate making it absolutely clear how the operation should be performed. Altogether there are thirty-two colored plates, and the elegantly printed pages comprise a masterly and thorough analysis of the whole subject.

Differential diagnosis is given elaborately, and the author maintains that there is only one remedy and that one is immediate and complete excision.

His exposition of the nature of this disease, which he says is strictly surgical, is so clear that he makes a convert to his views of treatment, of every one who reads the book.

